# 2298: Allergic asthma is associated with elevated sphingolipid levels in children

**DOI:** 10.1017/cts.2017.219

**Published:** 2018-05-10

**Authors:** Jennie G. Ono, Benjamin I. Kim, Tilla S. Worgall, Stefan Worgall

## Abstract

OBJECTIVES/SPECIFIC AIMS: To determine if altered sphingolipid metabolism and composition are associated with childhood-onset asthma. METHODS/STUDY POPULATION: Sphingolipid profiles and composition were analyzed in a pilot cohort of pediatric with asthma (n=22), and in nonasthmatic controls (n=17). The cohort includes males and females, ages 5–17 years with no prior history of asthma or wheezing, and those who have been previously diagnosed with asthma by a pediatric pulmonologist. Subjects who have a history of prematurity, chronic lung disease, acute respiratory infection, malignancy, autoimmune disorders, immunodeficiency, or sickle cell anemia were excluded. Asthma and nonasthma phenotypes were determined through clinical history, standardized asthma symptom checklists, medical record review and spirometry. Masses of sphingolipids were quantified by mass spectrometry (HPLC-MS/MS) in serum and exhaled breath condensates (EBC). Allergy status was determined through clinical questionnaire, blood IgE (>150 IU/mL) and blood eosinophils (>0.3×103/mcl). RESULTS/ANTICIPATED RESULTS: Multiple species of sphingolipids and ceramides were found to be higher in the serum and EBC of asthmatics compared with controls in the overall cohort. In serum, these species include C16 (*p*=0.05), C16DH (*p*=0.05), C18:1DH (*p*=0.002), C20 (*p*=0.05), Sphingosine (*p*=0.05), and S1P (*p*=0.04). In EBC, asthma was associated with higher levels of C18:1DH (*p*=0.05), C20 (*p*=0.05), C22 (*p*=0.05), Sphinganine (*p*=0.05), Sphingosine (*p*=0.04), and S1P (*p*=0.06). When data were stratified for allergic status, the increases in serum sphingolipids were largely associated with total IgE levels greater than 150 IU/mL. Sphingolipids which were increased in allergic asthma (n=13) compared with allergic controls (n=5) included C16 (*p*=0.006), C16DH (*p*=0.006), C18:1DH (*p*=0.06), C20 (*p*=0.048), C22 (*p*=0.02), C24 (*p*=0.02), C24:1 (*p*=0.02), Sphinganine (*p*=0.02), Sphingosine (*p*=0.01), and S1P (*p*=0.02). Notably, only C18:1DH remained increased in asthmatics regardless of allergic status, in both low and high total IgE subjects. DISCUSSION/SIGNIFICANCE OF IMPACT: Data from this pilot cohort suggest that sphingolipids are altered in asthmatic compared with nonasthmatic children, particularly in association with a history of allergy and elevated blood IgE. This trend was also demonstrated in exhaled breath condensate, suggesting that sphingolipids are altered both in serum and airway fluid. Only 1 species of sphingolipid measured, C18:1DH, was elevated in asthmatics regardless of allergic status. Notably, this sphingolipid was recently identified to be associated with exercise induced wheezing (EIW) and asthma persistence overtime, in a large case-control study of children with and without asthma (Perzanowski *et al*., in press). EIW has been identified as a specific phenotype of asthma, and can be present with or without allergy/atopy. Taken together, these data suggest that altered sphingolipids may contribute towards the underlying pathophysiology of asthma, the understanding of which can lead to improved characterization of asthma phenotypes.

**Reference** **Perzanowski M,** ***et al***. Distinct serum sphingolipid profiles among school-age children with exercise-induced wheeze and asthma persistence. *American Journal of Respiratory and Critical Care Medicine* 2017 (in press).

